# CBCT of Osteogenesis Imperfecta of the Inner Ear

**DOI:** 10.5334/jbr-btr.875

**Published:** 2015-12-30

**Authors:** Patrick Gillardin, Marc Lemmerling, F. M. Vanhoenacker, Dirk Dewilde, Adelard I. De Backer

**Affiliations:** 1 Department of Radiology, AZ Sint-Lucas, Groenebriel 1, B-9000 Gent, Belgium; 2 Department of Radiology, UZ Gasthuisberg, Belgium; 3 Department of Radiology, UZ Gent, Gent, Belgium; 4 Department of Radiology, UZ Antwerpen, Antwerpen, Belgium; 5 Department of Radiology, AZ Sint-Maarten, Duffel, Belgium

**Keywords:** Otosclerosis, Osteogenesis imperfecta

A 42-year-old female known with osteogenesis imperfecta (OI) was referred to our department with complaints of deteriorating hearing loss. The medical history, besides some limb fractures, secondary to the OI, was negative. During clinical examination, a sensorineural hearing loss was confirmed bilaterally.

A cone-beam computerized tomography (CBCT) was performed and revealed an otic capsule demineralization at the level of the antefenestral fissula (white arrows) and the pericochlear area (black arrows) on either side, suggestive of bilateral otosclerosis (Figure A). Other anatomic landmarks of the outer, middle and inner ear were normal. These findings are in line of OI type I involving in the temporal bone.

**Figure A:**
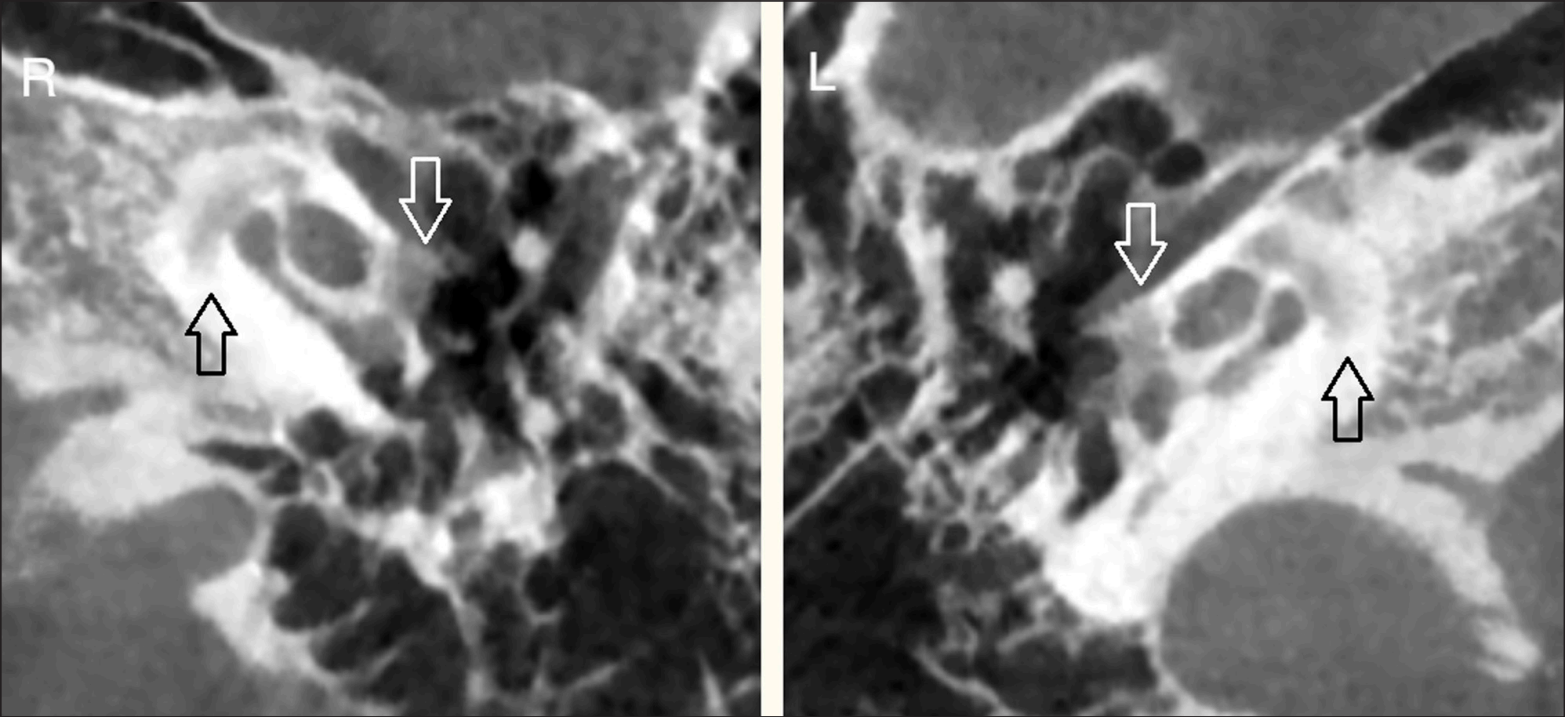


OI, or brittle bone disease, is a congenital hereditary disorder with many types, resulting in fracture-prone bone fragility. Due to an aberrant collagen type I synthesis, a predominant building block in the development of the extracellular matrix in most tissues, osteoporotic bone is formed. Other manifestations include ligamentous laxity, blue colored sclera, and as in this case, premature hearing loss. The incidence rate is approximately 1 in 12,000 births, with equal distribution between sexes. The most frequent form is type I, presenting with mild bone fragility, fractures, mild limb deformities in 50% of cases and hearing loss. Life expectancy depends on the type of OI, with type II (fetal/perinatal) having a lethal outcome. Mutations in the COL1A1 and COL1A2 genes are responsible for 90% of the cases. Multiple detector computed tomography (MDCT) is the golden standard to demonstrate demineralization of the otic capsule. The very high spatial resolution (125 µm), better image quality and lower radiation dose make CBCT a very tough competitor for MDCT. A schematic axial representation at the level of the incudomalleal joint is added depicting CBCT findings in OI compared to normal subjects (Figure B). Contrast-enhanced magnetic resonance imaging (MRI) may depict homogeneous enhancement of the otic capsule, possibly caused by leaking of contrast in the abnormal bone. The fundus of the internal auditory canal and the labyrinthine segment of the facial nerve can also enhance. T2 weighted images may reveal mural irregularities of the labyrinth and basal turn of the cochlea.

**Figure B:**
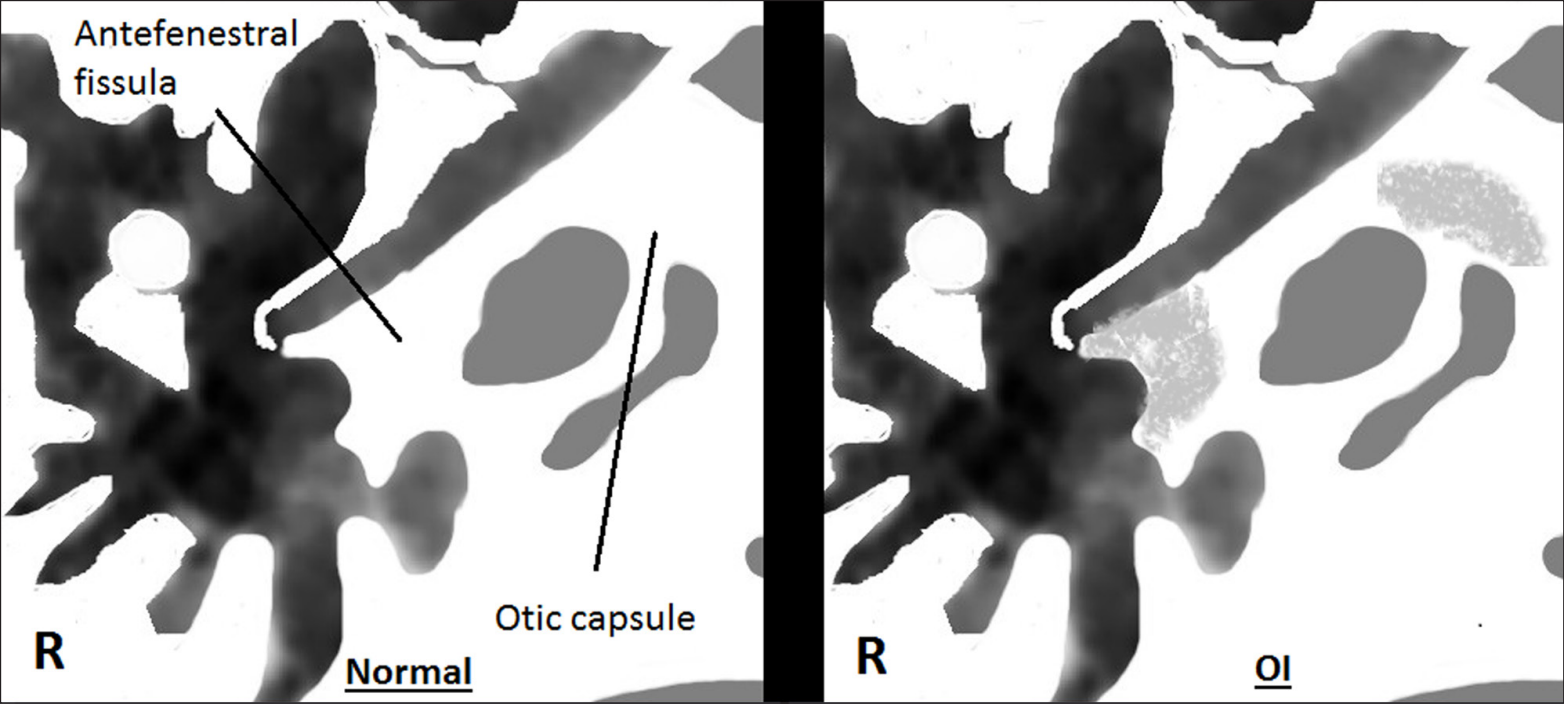


The differential diagnosis of bilateral otic capsule hypoattenuation in the temporal bone includes osteogenesis imperfecta, otosclerosis, otosyphilis, Paget’s disease and dysplastic bone diseases. As no cure exists for OI, treatment for bone involvement is symptomatic. Treatment options for hearing loss include bone-anchored hearing aids, cochlear implants and stapes surgery.

In conclusion, whenever a physician encounters a patient with osteogenesis imperfecta and hearing loss, a temporal manifestation of osteogenesis imperfecta must be kept in mind.

## Competing Interests

The authors declare that they have no competing interests.
